# Decidual Neutrophil Infiltration Is Not Required for Preterm Birth in a Mouse Model of Infection-Induced Preterm Labor

**DOI:** 10.4049/jimmunol.1302891

**Published:** 2014-02-05

**Authors:** Sara F. Rinaldi, Rob D. Catalano, Jean Wade, Adriano G. Rossi, Jane E. Norman

**Affiliations:** * Medical Research Council Centre for Reproductive Health, University of Edinburgh, Queen’s Medical Research Institute, Edinburgh EH16 4TJ, United Kingdom;; † Tommy’s Centre for Maternal and Fetal Health, University of Edinburgh, Queen’s Medical Research Institute, Edinburgh EH16 4TJ, United Kingdom; and; ‡ Medical Research Council Centre for Inflammation Research, University of Edinburgh, Queen’s Medical Research Institute, Edinburgh EH16 4TJ, United Kingdom

## Abstract

Parturition is associated with a leukocyte influx into the intrauterine tissues; however, the exact role these leukocytes play in the onset of labor remains unclear. Neutrophil infiltration of the uteroplacental tissues has been particularly associated with infection-associated preterm labor (PTL) in both women and mouse models. In this study, we investigated the role of neutrophils in a mouse model of infection-induced PTL. Intrauterine administration of LPS on day 17 of gestation resulted in a 7-fold increase in the number of decidual neutrophils compared with control mice receiving PBS (*p* < 0.01; *n* = 8–11). We hypothesized that neutrophil influx is necessary for PTL and that neutrophil depletion would abolish preterm birth. To test this hypothesis, mice were depleted of neutrophils by treatment with anti–Gr-1, anti–Ly-6G, or the appropriate IgG control Ab on day 16 of gestation prior to LPS on day 17 (*n* = 6–7). Successful neutrophil depletion was confirmed by flow cytometry and immunohistochemistry. Neutrophil depletion with Gr-1 resulted in reduced uterine and placental *Il-1β* expression (*p* < 0.05). Neutrophil depletion with Ly-6G reduced uterine *Il-1β* and *Tnf-α* expression (*p* < 0.05). However, neutrophil depletion with either Ab did not delay LPS-induced preterm birth. Collectively, these data show that decidual neutrophil infiltration is not essential for the induction of infection-induced PTL in the mouse, but that neutrophils contribute to the LPS-induced inflammatory response of the uteroplacental tissues.

## Introduction

Preterm labor (PTL), defined as labor prior to 37 wk gestation, is a major clinical problem estimated to affect between 5 and 18% of pregnancies worldwide annually. Preterm birth is the leading cause of neonatal mortality and morbidity, with >1 million babies dying each year due to complications related to their premature birth (1). Current treatment options for PTL are mainly limited to tocolytic agents, which aim to block myometrial contractility. However, these tocolytic drugs have proved to be largely ineffective at preventing preterm delivery, and there is little evidence that they actually improve neonatal outcome (2). Therefore, there is an urgent need for better treatment options.

The development of novel treatments for PTL is limited by the fact that the causes of PTL are often unclear. Intrauterine infection has been identified as a major cause of PTL, with up to 40% of preterm deliveries associated with the presence of an intrauterine infection (3). There is now strong evidence that term labor is an inflammatory event, with the onset of parturition at term being associated with increased production of inflammatory mediators, such as TNF-α, IL-1β, and IL-6 at the maternal–fetal interface, as well as an influx of leukocytes, mainly neutrophils and macrophages, into the cervix, myometrium, and fetal membranes (4–8). Given this strong link between the onset of parturition and inflammation, it is now believed that the presence of an intrauterine infection may prematurely activate inflammatory pathways, normally initiated at term, resulting in preterm delivery (9, 10). A clearer understanding of the underlying mechanisms surrounding the onset of PTL is crucial to the development of novel treatments that can effectively delay preterm delivery and ultimately improve neonatal outcome.

Although leukocyte influx has been identified as a key event occurring in coordination with the onset of parturition, the precise timing of the leukocyte infiltration and the role these immune cells play in the onset of labor remains relatively unclear. Additionally, there remains much debate as to whether these immune cells are a causative factor in the initiation of labor or if the primary role of these cells is in the repair and remodelling of the uterus and cervix following parturition (11–14).

Interestingly, Hamilton et al. (15) recently reported that neutrophil numbers were increased in the decidua of women in infection-associated PTL when compared with women in either idiopathic preterm or normal-term labor. A similar neutrophil influx has been report in a mouse model of infection-induced PTL, which was not observed in a model of noninfection-induced PTL (12, 16), suggesting that neutrophils may be particularly involved in infection-induced PTL.

The administration of mAbs in vivo to deplete specific cell types is a commonly used strategy to investigate the role of specific immune cells in various disease models, such as acute lung injury (17) and systemic infection (18). In recent years, in vivo depletion studies have also been used to investigate the role of specific immune cell populations in normal and adverse pregnancy outcomes (11, 19–22). However, to date, the role of neutrophils in a mouse model of LPS-induced PTL has not been examined. Therefore, in this study, we evaluated the effect of neutrophil depletion using two different mAbs on preterm delivery and the LPS-induced inflammatory response of the uteroplacental tissues. We hypothesized that neutrophils are necessary for the onset of LPS-induced PTL and that depletion of neutrophils would reduce the LPS-induced inflammatory response of the uteroplacental tissues and thus abolish preterm delivery.

## Materials and Methods

### Mouse model of infection-induced PTL

All animal studies were conducted under a U.K. Home Office license to J.E.N. (60/4241) in line with the Animals Scientific Procedure Act (1986). Timed-pregnant CD-1 mice were obtained from Charles River Laboratories (Margate, U.K.) on days 9–11 of gestation (the day vaginal plug was found was designated day 1 of gestation). Mice were acclimatized for a minimum of 6 d prior to surgery. On day 17 of gestation, mice were anesthetized with isoflurane (5% for induction and 2.5% for maintenance) in oxygen. A mini-laparotomy procedure was performed exposing the two uterine horns, and the number of viable fetuses in each horn was recorded. Using a 33-gauge Hamilton syringe, the horn with the most viable fetuses was injected with either LPS (1–20 μg LPS; from *Escherichia coli* 0111:B4; Sigma-Aldrich, Poole, U.K.) or sterile PBS (Life Technologies, Paisley, U.K.) each in a 25 μl volume. Injections were performed directly into the uterine lumen between the first and second anterior fetuses. Care was taken not to enter the amniotic cavity. The wound was then closed, and mice received a s.c. injection of Vetergesic analgesia (Alstoe, York, U.K.) at a dose of 0.03 mg/ml in 60 μl. Mice were kept at 30°C while they recovered from surgery, before being transferred to individual cages for continuous monitoring using individual closed-circuit television cameras and a digital video recorder. The time to delivery was recorded and defined as the number of hours from the time of intrauterine injection to delivery of the first pup.

In experiments to investigate the role of neutrophils in the induction of infection-induced PTL, Ab-based depletion was performed using either an anti–Gr-1 Ab or anti–Ly-6G Ab prior to intrauterine LPS administration. In Gr-1 experiments, on day 16 of gestation, mice received an i.p. injection of anti–Gr-1 (clone RB6-8C5; R&D Systems, Abingdon, U.K.) or IgG2b isotype control Ab (R&D Systems) at a dose of 250 μg in 500 μl. In Ly-6G experiments, on day 16 of gestation, mice received an i.p. injection of anti–Ly-6G (clone 1A8; BioLegend, Cambridge, U.K.) or IgG2a isotype control Ab (BioLegend) at a dose of 500 μg in 500 μl. Following Ab administration on day 16 of gestation, all mice received 20 μg intrauterine LPS on day 17 of gestation as described above.

### Blood and tissue collection

Mice were culled by lethal exposure to CO_2_ 6 h postsurgery. Blood and uteroplacental tissues were collected for analysis. Immediately after cull, blood was collected from the vena cava, and the uterine horns were dissected out. All pups were removed from the uterine horns and decapitated. Uterine tissue was sampled from three separate sites within the uterus, fetal membranes were dissected free from the placenta, and these tissues were collected from three separate gestational sacs. Uteroplacental tissues were stored for quantitative real-time PCR (qRT-PCR) analysis in RNA*later* (Sigma-Aldrich) at −80°C until processing. Uterine samples for immunohistochemistry were fixed in 4% neutral buffered formalin and embedded in paraffin blocks.

### Flow cytometry

Flow cytometry was used to confirm neutrophil depletion following anti–Gr-1 and anti–Ly-6G treatment. To prevent clotting, 20 μl whole blood was mixed with an equal volume of sodium citrate and kept on ice. Samples were incubated with PE-labeled rat anti-mouse CD45 Ab (1:100; BioLegend) and Pacific Blue–labeled rat anti-mouse Ly-6G Ab (1:100; BioLegend) for 30 min on ice. RBCs were then lysed, and the cells were fixed by the addition of 1 ml BD FACSlyse solution (BD Biosciences, Oxford, U.K.). Samples were centrifuged for 5 min at 350 × *g*, the supernatant removed, and the samples resuspended in 200 μl PBS for flow cytometric analysis. Prior to analysis, 50 μl Flow-Check fluorospheres (Beckman Coulter, High Wycombe, U.K.) was added to each sample to allow subsequent calculation of the absolute number of cells counted. Analysis was carried out using the BD LSR Fortessa (BD Biosciences), and data were collected using BD FACSDiva software (BD Biosciences) and analyzed using FlowJo software (Tree Star, Ashland, OR). Granulocytes, monocytes, and lymphocytes were gated based on their forward and side scatter. Neutrophils were specifically gated based on expression of both CD45 and Ly-6G.

### Immunohistochemistry

Immunohistochemistry was used to localize neutrophils in the uterus of mice. Tissue sections (5 μm) were dewaxed and rehydrated in ethanol. Endogenous peroxidase activity was blocked by incubating slides for 15 min in 3% hydrogen peroxidase. To block nonspecific binding, slides were incubated for 30 min at room temperature in 5% normal goat serum. Slides were then incubated overnight at 4°C with the primary Ab diluted in 5% normal goat serum (rat anti-mouse Gr-1, 1:1000, R&D Systems; or rat anti-mouse Ly-6G, 1:500, BioLegend). In negative control sections, the primary Ab was replaced with normal goat serum. All sections were then incubated with the secondary Ab (ImmPRESS anti-rat IgG reagent; Vector Laboratories, Peterborough, U.K.) for 30 min at room temperature, and positive staining was detected with 3,3-diaminobenzidine substrate for peroxidase for 5 min. Sections were counterstained in hematoxylin, dehydrated in ethanol, and mounted. Images were obtained using a PROVIS microscope (Olympus Optical, Hamburg, Germany) and AxioVision Rel 4.8 software (Zeiss, Cambridge, U.K.).

Neutrophil influx into the uterus 6 h postsurgery was quantified stereologically and compared between PBS and LPS treatment groups. The myometrium and decidua of each uterus section were analyzed separately. Each uterus section was tiled using a ×5 objective lens and Image-Pro Plus software. The area of interest (i.e., myometrium or decidua) was selected, and a total of 10 randomized fields of view were visualized using a ×40 objective lens. The number of positive cells per section was counted. In total, nine sections were counted per mouse, and the average number of positive cells counted per area was calculated for each mouse and averaged across treatments for both the decidua and myometrium.

### qRT-PCR

Total RNA was extracted from uterus, fetal membranes, and placental tissue collected 6 h postsurgery using the RNeasy mini kit (Qiagen, Crawley, U.K.) as per the manufacturer’s guidelines. Total RNA (300 ng) was reverse transcribed using the High Capacity cDNA Reverse Transcription kit (Applied Biosystems, Life Technologies). qRT-PCR was carried out to quantify the mRNA expression of specific genes of interest. Predesigned gene expression assays from Applied Biosystems were used to examine the expression of *Cxcl1* (Mm04207460_m1), *Cxcl2* (Mm00436450_m1), *Cxcl5* (Mm00436451_g1), *Il-1β* (Mm00434228_m1), the neutrophil marker neutrophil granule protein (*Ngp*; Mm01250218_m1), and *Tnf-α* (Mm99999068_m1). Primers and probes for *β-actin* and *Il-6* were designed using Primer Express Software Version 3 (Applied Biosystems). Details of designed *β-actin* and *Il-6* primer and probe sequences are given in Table I. Target gene expression was normalized for RNA loading using *β-actin*, and the relative expression in each sample was calculated relative to a calibrator sample (untreated day 18 uterus), which was included in all reactions, using the 2^−ΔΔ^ threshold cycle method of analysis. All qRT-PCR analysis was performed on an Applied Biosystems 7900HT instrument (Applied Biosystems).

### Statistical analysis

Data are presented as mean ± SEM. Time to delivery data were log transformed prior to analysis. Normally distributed data were then analyzed by one-way ANOVA followed by Dunnett multiple comparison test in which more than two treatment groups were present or unpaired *t* tests when only comparing between two treatments. All statistical analyses were performed using GraphPad Prism 5.0 software (GraphPad, San Diego, CA). A *p* value <0.05 was considered statistically significant.

## Results

### Intrauterine LPS administration dose-dependently induces preterm delivery

**FIGURE 1.**
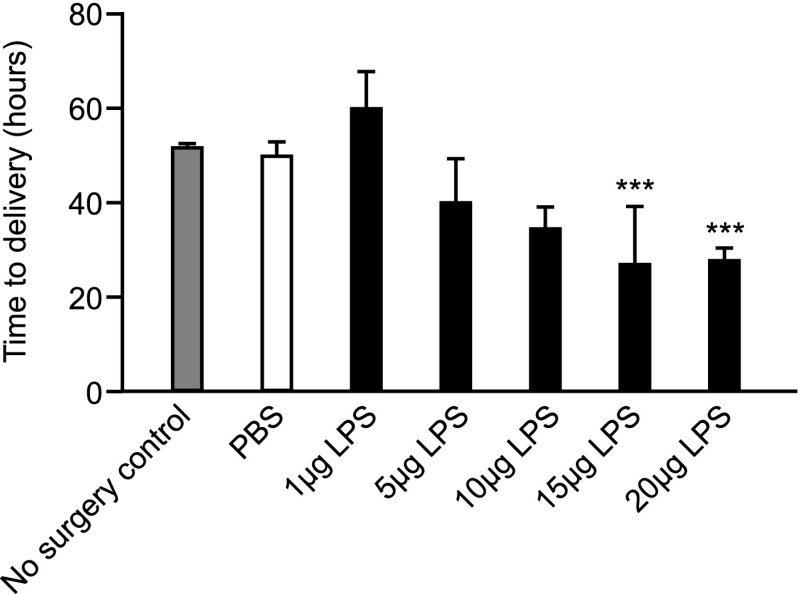
Effect of intrauterine LPS administration on time to delivery. Time to delivery was determined in mice receiving intrauterine injection of either PBS, 1, 5, 10, 15, or 20 μg LPS (*n* ≥ 5 in each treatment group). Time to delivery was also monitored in a group of mice receiving no surgery as a control (*n* = 8). Data presented as mean ± SEM (error bars). Data analyzed by one-way ANOVA followed by Dunnett post hoc analysis. ***p < 0.001 compared with PBS.

To determine the optimum dose of LPS to use in our model that could reliably induce preterm delivery, a dose-response experiment was performed. Time to delivery was also assessed in a group of mice that did not undergo surgery to assess the impact of surgery alone on time to delivery. There was no difference in the time to delivery of the untreated control compared with mice undergoing a laparotomy and being treated with intrauterine PBS (mean time to delivery of no surgery control: 51.34 ± 1.13 [SEM] versus PBS mean time to delivery: 49.58 ± 3.71 h; Fig. 1). Intrauterine LPS administration dose-dependently reduced the time to delivery, with mice treated with both 15 and 20 μg delivering significantly earlier compared with the PBS control group (15 μg LPS mean time to delivery: 26.44 ± 18.09 h, *p* < 0.001 compared with PBS control group; 20 μg LPS mean time to delivery: 27.23 ± 3.37 h, *p* < 0.001 compared with PBS control group; Fig. 1). Subsequent experiments were performed with 20 μg LPS, as this dose resulted in reliable preterm delivery with the least variation in time to delivery between mice.

### Intrauterine LPS administration increases chemokine expression in the uteroplacental tissues and induces a neutrophil influx into the decidua

**FIGURE 2.**
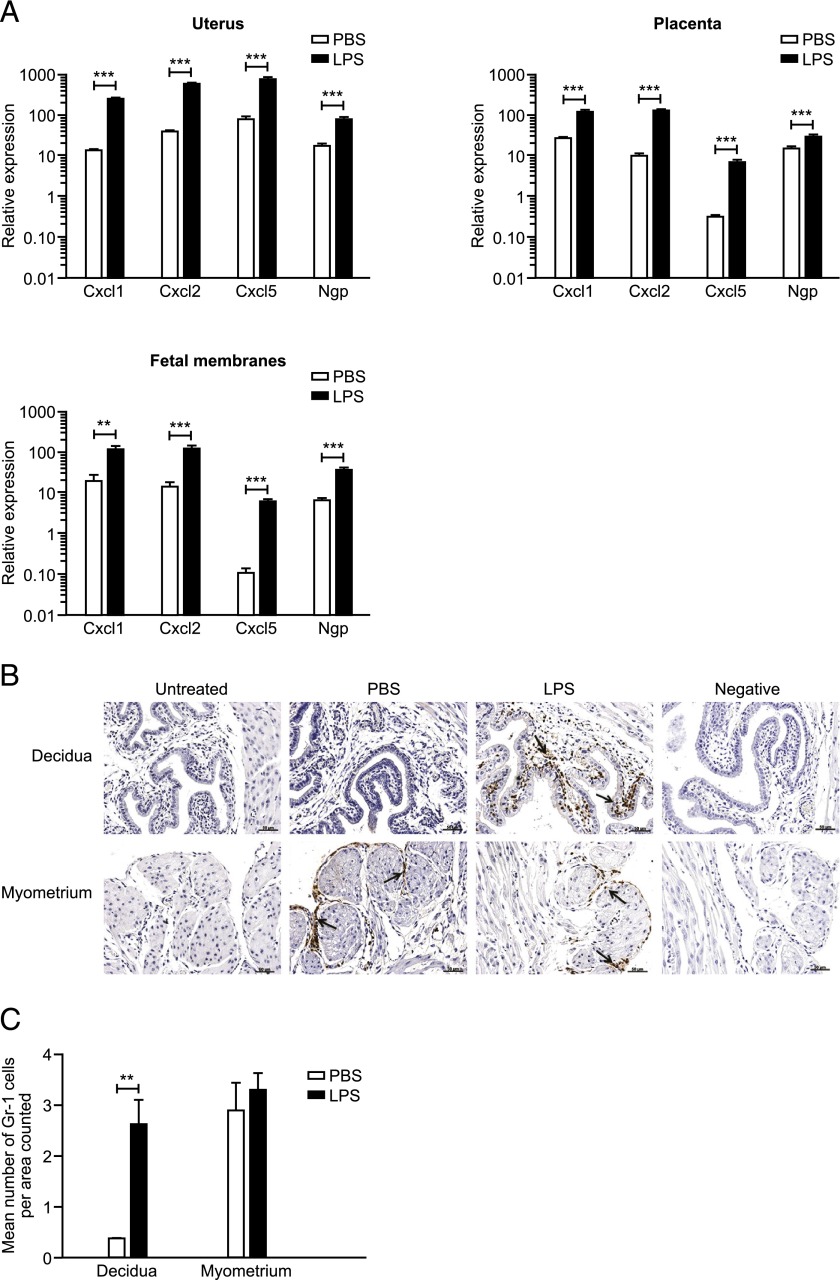
Effect of intrauterine LPS administration on chemokine mRNA expression and neutrophil influx into the uteroplacental tissues. Uterus, placenta, and fetal membranes were collected 6 h postsurgery from mice receiving PBS (*n* = 8) or 20 μg LPS (*n* = 11). mRNA expression of specific chemokines and a neutrophil marker was quantified by qRT-PCR. (**A**) Expression of the neutrophil chemokines *Cxcl1*, *Cxcl2*, and *Cxcl5* and the neutrophil marker *Ngp* in the uterus, placenta, and fetal membranes. (**B**) Representative images of neutrophil localization using immunohistochemical staining for Gr-1. Scale bars, 50 μm. All images taken with a ×20 objective lens. (**C**) Quantification of Gr-1–positive cells in the uterus. Data presented as mean ± SEM (error bars). Data analyzed by *t* test. **p < 0.01, ***p < 0.001 compared with PBS.

To investigate the effect of intrauterine LPS administration on neutrophil infiltration to the uteroplacental tissues, the mRNA expression of the neutrophil chemokines *Cxcl1*, *Cxcl2*, and *Cxcl5* and the neutrophil marker *Ngp* were examined in the uterus, placenta, and fetal membranes using qRT-PCR 6 h postsurgery. Details of designed primer and probe sequences are given in Table 1. In the uterus and placenta, LPS treatment significantly increased the expression of all three neutrophil chemokines and the neutrophil marker *Ngp* compared with mice receiving PBS (*p* < 0.001; Fig. 2A). Similarly, in the fetal membranes, expression of *Cxcl1* (*p* < 0.01), *Cxcl2*, *Cxcl5*, and *Ngp* (*p* < 0.001) was also elevated in mice treated with LPS compared with those receiving PBS (Fig. 2A).

**Table I.** Primer and probe sequences designed using Primer Express software

Gene	Primer/Probe	Sequence
β-actin	Forward	5′-GCTTCTTTGCAGCTCCTTCGT-3′
	Reverse	5′-GCGCAGCGATATCGTCATC-3′
	Probe	5′-CACCCGCCACCAGTTCGCCAT-3′
Il-6	Forward	5′-CCACGGCCTTCCCTACTTC-3′
	Reverse	5′-TGCACAACTCTTTTCTCATTCCA-3′
	Probe	5′-TCACAGAGGATACCACTCCCAACAGACCTG-3′

To confirm that the LPS-induced increased chemokine and *Ngp* expression was due to neutrophil influx into the uterus and to determine the site of recruitment of neutrophils in the uterus, we performed immunohistochemistry with an anti–Gr-1 Ab on uterine tissue collected 6 h postsurgery from mice who had received either PBS or LPS or from untreated mice on day 17 of gestation, as a comparator.

On day 17 of gestation, untreated mice that did not undergo surgery had very few, if any, neutrophils present in the myometrium and decidua (Fig. 2B). PBS-treated mice had positive Gr-1 staining in the connective tissue layer surrounding the longitudinal myometrial muscle bundles and within blood vessels of the myometrium, with very few if any neutrophils in the decidua. In contrast, in mice treated with intrauterine LPS, Gr-1–positive neutrophils were also localized within the myometrium, but there was an additional large influx of neutrophils into the decidua. Quantification of the Gr-1–positive cells within the uterine tissue confirmed that LPS treatment induced a significant 7-fold increase in decidual neutrophil density (*p* < 0.01) compared with mice receiving PBS (Fig. 2C). There was no difference between the treatment groups in neutrophil density in the myometrium.

### Administration of anti–Gr-1 on day 16 of gestation successfully depletes circulating neutrophils

**FIGURE 3.**
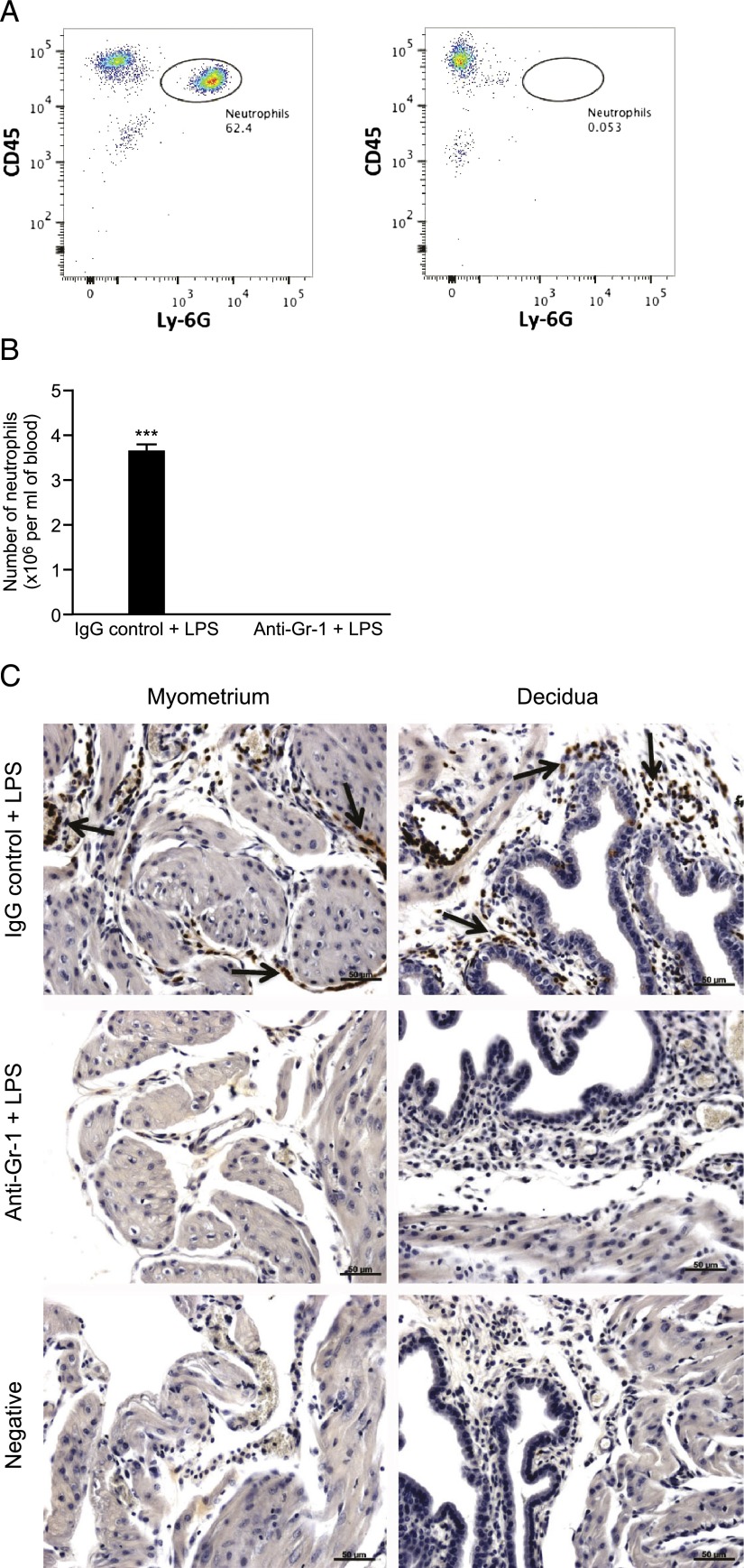
Depletion of neutrophils using anti–Gr-1. Mice were treated with either anti–Gr-1 Ab (*n* = 7) or an IgG control Ab (*n* = 6) prior to intrauterine LPS administration, and maternal blood and uterine tissue were collected 6 h post–LPS administration. (**A**) Flow cytometric analysis of maternal blood. Cells were gated based on expression of CD45 and Ly-6G. Representative flow plots from one mouse treated with the IgG control Ab (*left panel*) and one mouse treated with anti–Gr-1 Ab (*right panel*). The number given beside each gated region indicates the percentage of neutrophils counted out of the total number of live cells. (**B**) The total number of neutrophils per milliliter of blood. (**C**) Representative images of neutrophil localization by immunohistochemistry for Gr-1. Scale bars, 50 μm. All images taken with a ×20 objective lens. Data presented as mean ± SEM. Data analyzed by *t* test. ***p < 0.001.

To investigate whether the observed neutrophil influx was necessary for either induction of LPS-induced PTL or the LPS-induced inflammatory response in the uteroplacental tissues, we used Ab depletion to remove neutrophils prior to intrauterine LPS administration. The anti–Gr-1 Ab (RB6-8C5) is commonly used to deplete neutrophils in in vivo models (23, 24). Mice were treated with either anti–Gr-1 or the appropriate IgG control Ab on day 16 of gestation, prior to intrauterine LPS administration of day 17. Flow cytometry analysis of blood collected 6 h post–LPS administration confirmed that administration of the Gr-1 Ab successfully depleted circulating neutrophils (Fig. 3A, *right panel*), with significantly fewer circulating neutrophils seen in mice treated with anti–Gr-1 + LPS compared with those receiving IgG control + LPS treatment (mean number of neutrophils per milliliter of blood in IgG control group: 3.67 × 10^6^ ± 0.21 versus 0.0063 × 10^6^ ± 0.0018 in anti–Gr-1 group, *p* < 0.001; Fig. 3B). Immunohistochemistry confirmed that the Gr-1–positive neutrophils observed in the myometrium and decidua of control mice (IgG + LPS treated) were not present in mice treated with anti–Gr-1 + LPS (Fig. 3C).

### Depletion of neutrophils using anti–Gr-1 does not delay LPS-induced PTL, but does alter the inflammatory response of the uteroplacental tissues to LPS

**FIGURE 4.**
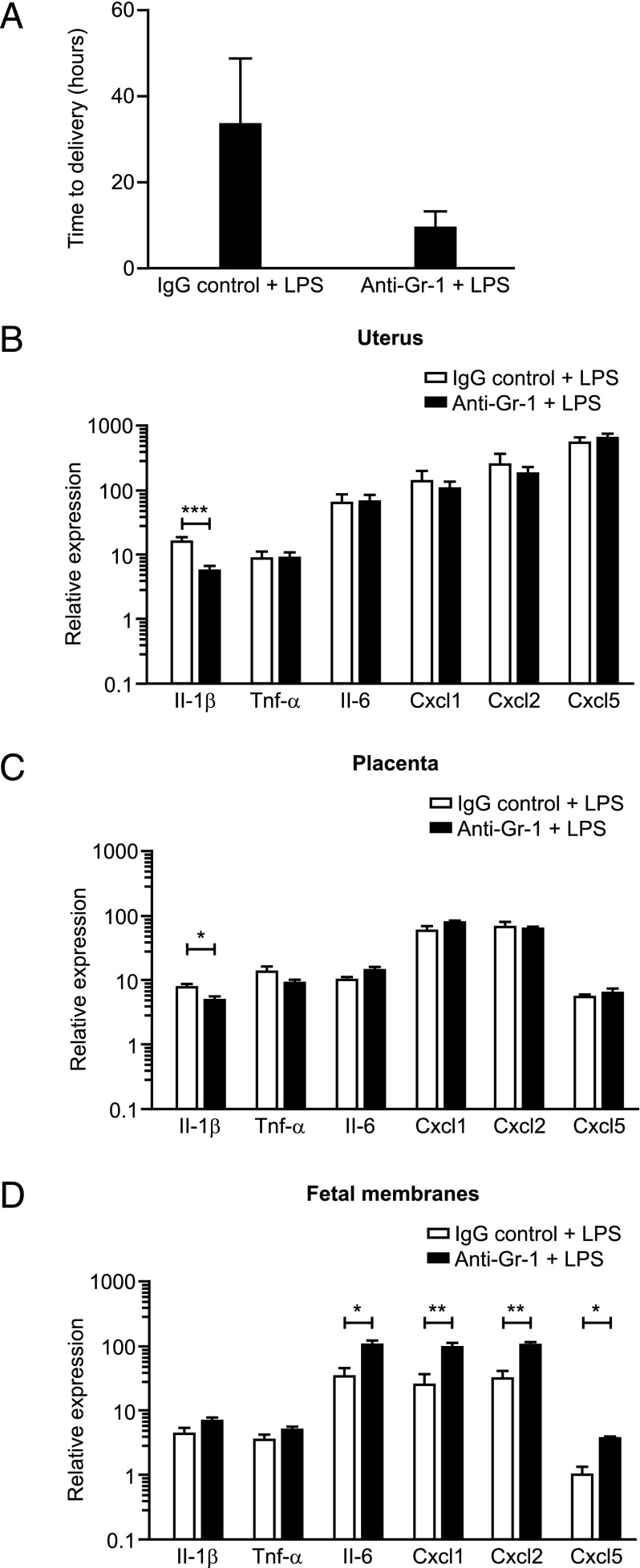
Effect of neutrophil depletion using anti–Gr-1 on LPS-induced preterm delivery and inflammatory gene expression in the uteroplacental tissues. (**A**) Time to delivery was determined in mice treated with either the IgG control Ab + LPS (*n* = 5) or anti–Gr-1 Ab + LPS (*n* = 5). Data presented as mean ± SEM (error bars), nonsignificant between the two groups. (**B–D**) Expression of inflammatory genes was assessed by qRT-PCR in uterus, placenta, and fetal membranes harvested 6 h postsurgery from mice treated with either the IgG control Ab + LPS (*n* = 5) or anti–Gr-1 Ab + LPS (*n* = 5). Data presented as mean fold change ± SEM (error bars). Data analyzed by *t* test. *p < 0.05, **p < 0.01, ***p < 0.001 compared with control.

There was no delay in the time to delivery in the Gr-1–treated mice compared with the control group. Indeed, we observed a nonsignificant trend toward earlier delivery in the anti–Gr-1 + LPS group (mean time to delivery: 8.95 h ± 1.91) compared with mice treated with IgG control + LPS (mean time to delivery 33.3 h ± 7.99, *p* = 0.12; Fig. 4A).

In contrast, treatment with anti–Gr-1 + LPS significantly reduced expression of *Il-1β* in the uterus and placenta compared with mice treated with IgG control + LPS (*p* < 0.001 and *p* < 0.05, respectively; Fig. 4B, 4C). In the fetal membranes, anti–Gr-1 + LPS treatment resulted in elevated expression of *Il-6* (*p* < 0.05), *Cxcl1* (*p* < 0.01), *Cxcl2* (*p* < 0.01), and *Cxcl5* (*p* < 0.05) compared with the control group (Fig. 4D).

### Administration of anti–Ly-6G on day 16 of gestation successfully depletes circulating neutrophils

**FIGURE 5.**
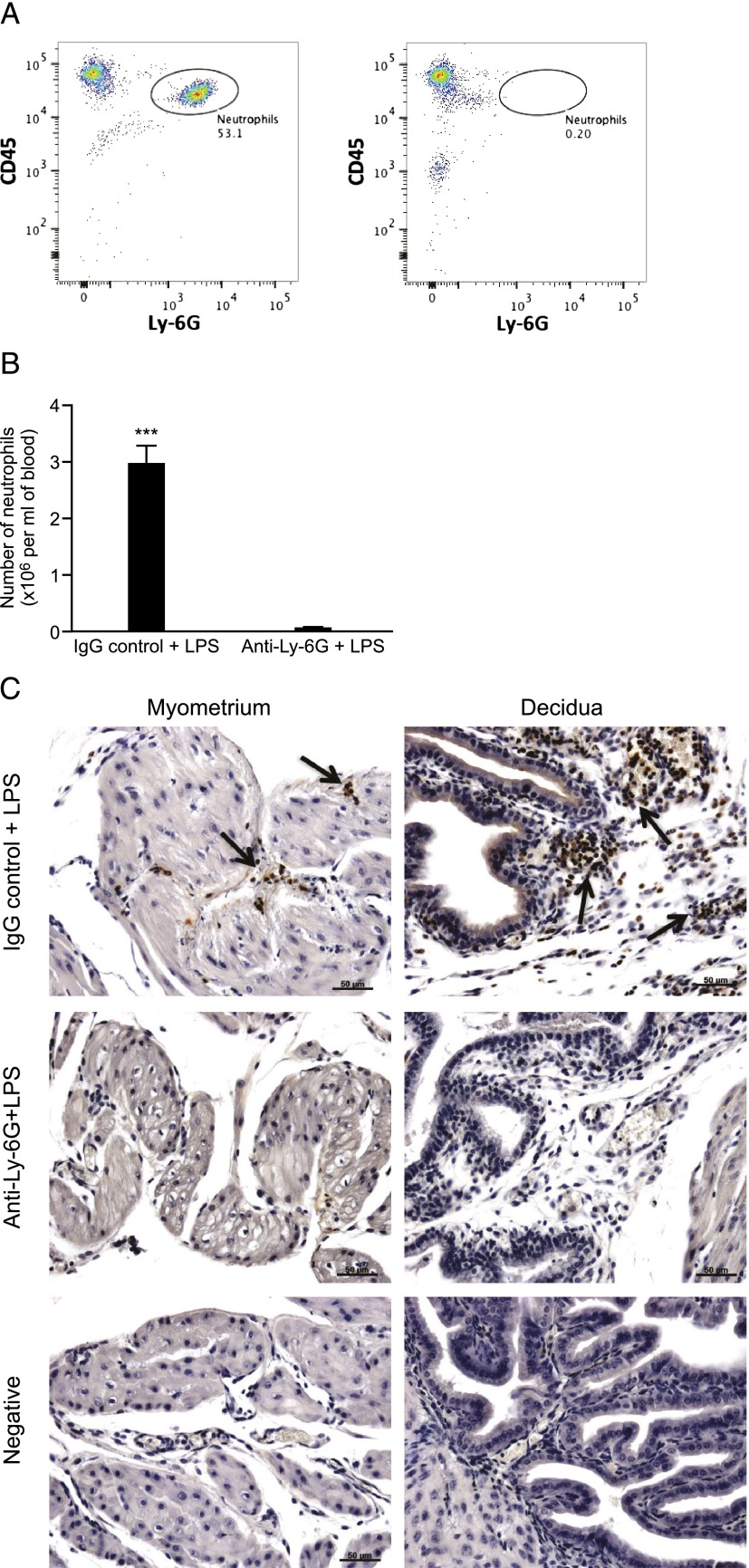
Depletion of neutrophils using anti–Ly-6G. Mice were treated with either anti–Ly-6G Ab (*n* = 7) or an IgG control Ab (*n* = 7) prior to intrauterine LPS administration, and maternal blood and uterine tissue were collected 6 h post-LPS administration. (**A**) Flow cytometric analysis of maternal blood. Cells were gated based on expression of CD45 and Ly-6G. Representative flow plots from one mouse treated with the IgG control Ab (*left panel*) and one mouse treated with anti–Ly-6G Ab (*right panel*). The number given beside each gated region indicates the percentage of neutrophils counted out of the total number of live cells. (**B**) The total number of neutrophils per milliliter of blood. (**C**) Representative images of neutrophil localization by immunohistochemistry for Ly-6G. Scale bars, 50 μm. All images taken with a ×20 objective lens. Data presented as mean ± SEM. Data analyzed by *t* test. ***p < 0.001.

As previous studies have demonstrated that administration of the anti–Gr-1 Ab can also deplete subsets of other immune cells including monocytes, T cells, and dendritic cells (25–27), we next investigated neutrophil depletion using the more specific anti–Ly-6G Ab. Mice were treated with either anti–Ly-6G or the appropriate IgG control Ab on day 16 of gestation, prior to intrauterine LPS administration on day 17. Flow cytometry analysis of blood collected 6 h post–LPS administration confirmed that administration of the Ly-6G Ab resulted in successful depletion of circulating neutrophils (Fig. 5A, *right panel*), with a significant reduction in the number of circulating neutrophils in mice treated with anti–Ly-6G + LPS compared with mice receiving IgG control + LPS (mean number of neutrophils per milliliter of blood in IgG control group: 2.95 × 10^6^ ± 0.35 versus 0.04 × 10^6^ ± 0.02 in the anti–Ly-6G group, *p* < 0.001; Fig. 5B). Immunohistochemical staining for Ly-6G confirmed that pretreatment with anti–Ly-6G prior to intrauterine LPS prevented the LPS-induced decidual neutrophil influx observed in control animals (Fig. 5C).

### Depletion of neutrophils using anti–Ly-6G does not delay LPS-induced PTL, but does alter the inflammatory response of the uteroplacental tissues to LPS

**FIGURE 6.**
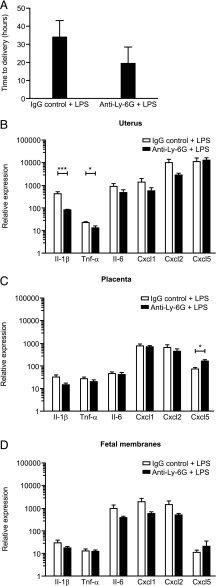
Effect of neutrophil depletion using anti–Ly-6G on LPS-induced preterm delivery and inflammatory gene expression in the uteroplacental tissues. (**A**) Time to delivery was determined in mice treated with either the IgG control Ab + LPS (*n* = 8) or anti–Ly-6G Ab + LPS (*n* = 8). Data presented as mean ± SEM (error bars). (**B–D**) Expression of inflammatory genes was assessed by qRT-PCR in uterus, placenta, and fetal membranes harvested 6 h postsurgery from mice treated with either the IgG control Ab + LPS (*n* = 7) or the anti–Ly-6G Ab + LPS (*n* = 6). Data presented as mean fold change ± SEM (error bars). Data analyzed by *t* test. *p < 0.05, ***p < 0.001 compared with control.

The mean time to delivery in mice treated with anti–Ly-6G + LPS was not found to be significantly different from IgG control + LPS controls (mean time to delivery in anti–Ly-6G group: 19.25 h ± 9.35 versus mean time to delivery in the IgG control group: 33.47 h ± 9.58, *p* = 0.14; Fig. 6A).

Pretreatment with anti–Ly-6G prior to LPS resulted in significantly reduced uterine expression of *Il-1β* and *Tnf-α* (*p* < 0.001 and *p* < 0.05, respectively) compared with the mice receiving the IgG control Ab prior to intrauterine LPS (Fig. 6B). In contrast, placental *Cxcl5* expression was significantly elevated in the anti–Ly-6G + LPS group compared with controls (*p* < 0.05; Fig. 6C). There was no effect of treatment on gene expression in the fetal membranes (Fig. 6D).

### Effect of neutrophil depletion using either anti–Gr-1 or anti–Ly-6G on the proportion of circulating monocytes and lymphocytes

**FIGURE 7.**
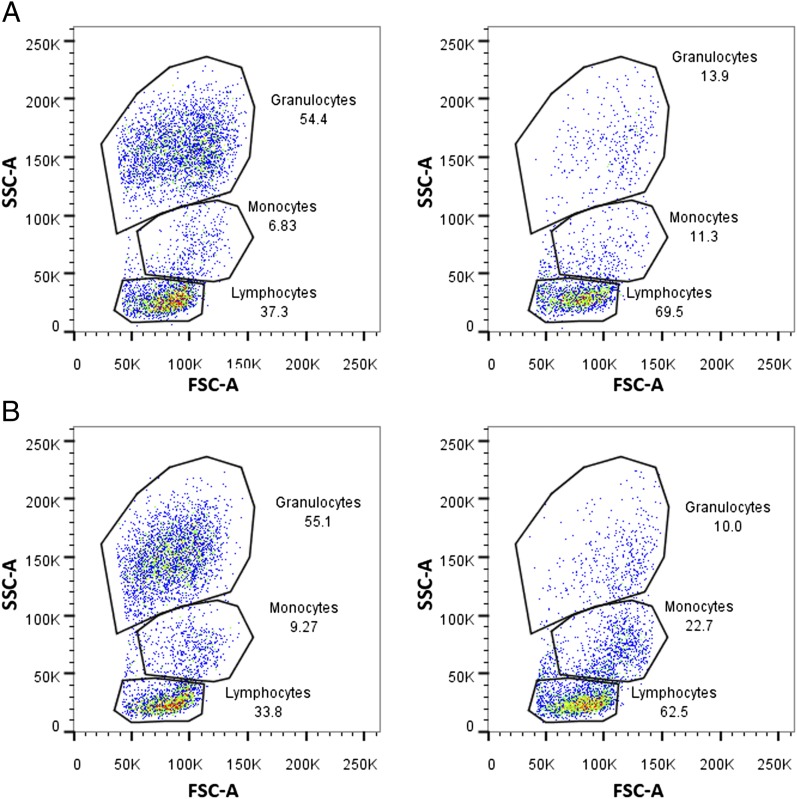
Effect of neutrophil depletion on circulating granulocyte, monocyte, and lymphocyte populations. Flow cytometric analysis of maternal blood collected 6 h post–LPS administration. Lymphocytes, monocytes, and granulocytes were gated based on their forward light scatter (FSC) and side scatter (SSC) profiles. (**A**) Gr-1 depletion: representative flow plots from one mouse treated with the IgG control Ab (*left panel*) and one mouse treated with anti–Gr-1 Ab (*right panel*). (**B**) Ly-6G depletion: representative flow plots from one mouse treated with the IgG control Ab (*left panel*) and one mouse treated with anti–Ly-6G Ab (*right panel*). The number given beside each gated region indicates the percentage of cells out of total leukocytes.

To assess whether neutrophil depletion with either Ab affected the proportion of circulating monocytes or lymphocytes, flow cytometry analysis was performed on maternal blood collected 6 h post–LPS administration. Granulocytes, monocytes, and lymphocytes were gated based on their forward and side scatter in mice treated with isotype control Ab (Fig. 7A, *left panel*) or anti–Gr-1 (Fig. 7A, *right panel*) or isotype control Ab (Fig. 7B, *left panel*) or anti–Ly-6G (Fig. 7B, *right panel*). The percentages of circulating granulocytes, monocytes, and lymphocytes as a percentage of the total leukocytes are given in Table II. As a result of neutrophil depletion, there was a large reduction in the percentage of circulating granulocytes [in which Ly-6G–positive neutrophils were almost completely depleted (Figs. 3, 5)] and consequently an increase in the overall proportion of circulating monocytes and lymphocytes (Table II). The proportions of circulating lymphocytes and monocytes as a percentage of the total mononuclear cells are given in Table III. When examining the effect of neutrophil depletion specifically on mononuclear cells, there was a small reduction in the proportion of circulating monocytes in response to Gr-1 depletion compared with the appropriate IgG + LPS control group, which was not observed in Ly-6G–treated mice (Table III).

**Table II.** The percentages of circulating lymphocytes, monocytes, and granulocytes following neutrophil depletion

	Gr-1 Depletion		Ly-6G Depletion	
	IgG + LPS (*n* = 6)	Anti–Gr-1 + LPS (*n* = 7)	IgG + LPS (*n* = 7)	Anti–Ly-6G + LPS (*n* = 7)
Percent of circulating lymphocytes/total leukocytes	35.65 ± 1.78	71.41 ± 5.34	33.89 ± 3.07	58.9 ± 4.64
Percent of circulating monocytes/total leukocytes	7.37 ± 0.59	10.93 ± 1.91	9.35 ± 0.45	20.24 ± 4.08
Percent of circulating granulocytes/total leukocytes	54.73 ± 2.46	14.37 ± 5.34	55.2 ± 3.20	16.69 ± 4.19

Data presented as mean ± SEM. Flow cytometric analysis of maternal blood collected 6 h post–LPS administration from mice treated with anti–Gr-1, anti–Ly-6G, or the appropriate isotype control Ab, prior to intrauterine LPS. Lymphocytes, monocytes, and granulocytes were gated based on their forward and side scatter profiles, and the proportion of circulating granulocytes, monocytes, and lymphocytes as a percentage of the total leukocytes was calculated.

**Table III.** Circulating mononuclear cell proportions following neutrophil depletion

	Gr-1 Depletion		Ly-6G Depletion	
	IgG + LPS (*n* = 6)	Anti–Gr-1 + LPS (*n* = 7)	IgG + LPS (*n* = 7)	Anti–Ly-6G + LPS (*n* = 7)
Percent of lymphocytes/total mononuclear cells	82.91 ± 1.08	86.33 ± 2.42	77.85 ± 1.59	74.63 ± 4.56
Percent of monocytes/total mononuclear cells	17.09 ± 1.08	13.67 ± 2.41	22.15 ± 1.59	25.37 ± 4.56

Data presented as mean ± SEM. Flow cytometric analysis of maternal blood collected 6 h post-LPS administration from mice treated with anti–Gr-1, anti–Ly-6G, or the appropriate isotype control Ab, prior to intrauterine LPS. Lymphocytes and monocytes were gated based on their forward and side scatter, and the proportions of circulating lymphocytes and monocytes as a percentage of the total mononuclear cells were calculated.

## Discussion

Although leukocyte infiltration into the uteroplacental tissues is known to occur in association with the onset of parturition, the precise role these immune cells play, and particularly the role they play in infection-induced preterm labor, remains unclear. In this study, we have shown that the presence of bacterial LPS, as a model of intrauterine infection, induces increased expression of the neutrophil chemokines *Cxcl1*, *Cxcl2*, and *Cxcl5* in the uteroplacental tissues and a neutrophil influx into the decidua. Using Ab-mediated neutrophil depletion with two separate Abs, we demonstrate that neutrophils are not required for preterm birth (at least in a mouse model of LPS-induced PTL), but that neutrophils contribute to the LPS-induced inflammatory response of the uteroplacental tissues.

Recent microarray analysis has identified that some of the most highly upregulated genes in human myometrium and cervix during labor are chemokines, including CXCL1, CXCL2, CXCL5, and CXCL8 (28), all of which are known to be strong neutrophil chemoattractants. Hamilton et al. (29) also recently identified that infection-associated (compared with idiopathic) PTL is associated with a distinct chemokine profile in the chorio-decidua, with a general downregulation of CC chemokines and elevated expression of a number of CXC chemokines, such as CXCL1, CXCL5, and CXCL8, in infection-associated PTL. Similarly, several studies have reported elevated expression of the mouse homologs *Cxcl1*, *Cxcl2*, and *Cxcl5* in uterine tissue collected from models of LPS-induced PTL (12, 30, 31). In agreement with this previous work, we found that intrauterine LPS administration significantly elevated the expression of all three neutrophil chemokines in the uterus, placenta, and fetal membranes. Collectively, these data suggest that in response to an intrauterine infection, the uteroplacental tissues increase their neutrophil chemotactic activity. We hypothesized that this results in increased neutrophil trafficking to the uterus and plays a crucial role in the onset of LPS-induced PTL. Surprisingly, however, neutrophil depletion had no effect on time to delivery in LPS-induced PTL, refuting this hypothesis.

In women, neutrophils are proposed to play an important role in cervical ripening and have been shown to infiltrate the myometrium and cervix in association with labor (7, 8). Additionally, it has been reported that women with infection-associated PTL have more neutrophils in the decidua, compared with women in idiopathic PTL or normal-term labor (15). Animal studies support these findings, demonstrating that neutrophil influx of the intrauterine tissues may be particularly involved in infection-associated PTL, but not PTL induced by progesterone withdrawal (12, 16). To investigate neutrophil influx in our model of infection-induced PTL, we measured expression of a neutrophil marker, *Ngp*, in the uteroplacental tissues. Additionally, we localized neutrophils using immunohistochemistry. We observed that intrauterine administration of LPS on day 17 of gestation increased mRNA expression of *Ngp* in the uterus, placenta, and fetal membranes within 6 h, and that this increased expression was coincident with a large neutrophil influx into the decidua in response to intrauterine LPS treatment, confirming a suggested role for neutrophils in infection-induced PTL. These data contrast to those of Menzies et al. (32), who demonstrated that expression of *Ngp* remains unchanged in laboring compared with nonlaboring mouse uterus and provide further support for the theory that infection-induced PTL and term labor may occur via different mechanisms, with neutrophils playing a specific role in infection-associated PTL (16, 33).

Interestingly, we also observed neutrophil infiltration in uterine tissue collected from PBS-treated mice. These neutrophils were localized to the connective tissue layer surrounding the longitudinal myometrial muscle bundles and were absent in untreated mice that did not undergo surgery. This finding suggests that the surgery itself induced a local inflammatory response and immune cell influx to the uterus. Shynlova et al. (34) also recently observed neutrophil infiltration into the uterus of mice receiving sham surgery in a model of artificial stretch, suggesting that uterine surgery during pregnancy can induce a rapid neutrophil response. However, decidual neutrophil infiltration was only observed in LPS-treated mice.

To investigate whether the observed decidual neutrophil infiltration was necessary for the onset of infection-induced preterm labor, we used two separate Abs to deplete neutrophils prior to intrauterine LPS administration. The anti–Gr-1 Ab (clone RB6-8C5) has been widely used to deplete neutrophils in mouse models to investigate the role they play in inflammatory pathologies (23, 24). This Ab recognizes two Ags expressed on the surface of some immune cells: Ly-6G and Ly-6C (35). Although Ly-6G expression is restricted to murine neutrophils, Ly-6C is also expressed on subpopulations of monocytes, macrophages, dendritic cells, and T cells (36–38). Several studies have reported that administration of anti–Gr-1 can also result in depletion of these other immune cell populations, thus making the specific role of neutrophils difficult to interpret (25–27). For this reason, many studies now focus on using the anti–Ly-6G–specific mAb (clone 1A8) to investigate the role of neutrophils, as this Ab has been shown to selectively deplete neutrophils while preserving other immune cell populations (27, 39, 40).

Previous studies investigating the effect of neutrophil depletion on normal and adverse pregnancy outcomes have been restricted to neutrophil depletion with the anti–Gr-1 Ab. These studies found that neutrophil depletion had no effect either on normal-term labor (11) or on models of CpG oligodeoxynucleotide-induced PTL in IL-10 knockout (19) or NK cell–deficient NOD mice (21). Our study is the first, to our knowledge, to investigate the effect of neutrophil depletion in a model of LPS-induced PTL and the first study to use the more specific anti–Ly-6G Ab. In agreement with these previous studies, although both flow cytometry and immunohistochemical analysis confirmed that administration of either anti–Gr-1 or anti–Ly-6G on day 16 of gestation successfully depleted circulating neutrophils, neutrophil depletion (using either Ab) did not prevent LPS-induced preterm birth. These data contrast with our original hypothesis. Indeed, we observed a nonsignificant trend toward earlier delivery in our neutrophil-depleted (compared with control) groups. These results suggest that although LPS-induced PTL is preceded by a large decidual neutrophil influx, neutrophils are not required for the preterm birth.

It is possible that neutrophils may play a different role in the onset of labor depending on the degree of infection present. However, as our dose-response data (and those of others) show, 20 μg LPS was the only dose that reliably induced preterm delivery with minimal variation (41). Studies to investigate whether neutrophils may be required for the onset of labor in response to lower doses of LPS would therefore require a large sample size to address variability in response.

If these infiltrating neutrophils are not involved in inducing PTL, then the question remains as to what role they have. Our results suggest that the neutrophils may ameliorate the effects of LPS, given that neutrophil-depleted mice delivered somewhat earlier than control mice. Interestingly, several studies have reported that neutrophil depletion actually results in increased susceptibility to infection and poorer outcomes in other models of infection-induced pathologies (42, 43), thus neutrophils may primarily be involved in controlling the LPS-induced inflammatory response. It is also possible that the primary role of the labor-associated neutrophil infiltration may be in preparation for postpartum repair and remodelling of the uterus and cervix, as has been previously suggested (12, 16, 44). Support for this hypothesis comes from a study that reported that neutrophil depletion using anti–Gr-1 significantly delayed endometrial repair in a mouse model of menstruation (23), indicating the important role that neutrophils play in repair and remodeling following other inflammatory reproductive processes. Further studies using repeated administration of the Abs to maintain neutrophil depletion into the postpartum period are required to test this hypothesis in our model.

Several studies have identified that leukocytes infiltrating the uteroplacental tissues during labor are a major source of inflammatory mediators, such as IL-1β, TNF-α, IL-6, IL-8, and matrix metalloproteinase-9 (6, 45, 46), suggesting that these immune cells are likely to contribute to the inflammatory response surrounding parturition. Our results are in agreement with these studies, as we observed altered LPS-induced inflammatory gene expression in the uteroplacental tissues when comparing depleted mice to control mice. Therefore, although neutrophils appear not to be required for the induction of LPS-induced PTL, these data suggest that they do contribute to the LPS-induced inflammatory response of the uteroplacental tissues. Interestingly, although depletion using the anti–Gr-1 Ab resulted in reduced *Il-1β* expression in the uterus and placenta, we observed increased expression of *Il-6*, *Cxcl1*, *Cxcl2*, and *Cxcl5* in the fetal membranes, giving further support to the suggestion that the Gr-1–positive cells that were depleted from the fetal membranes may be involved in inhibiting the LPS-induced inflammatory response. Fewer significant inflammatory gene expression changes were observed in response to depletion using the more specific anti–Ly-6G Ab compared with depletion using anti–Gr-1, and this may reflect that different immune cell populations are being depleted with the different Abs as previously reported in other models (25–27). Our data suggest that neutrophil depletion with the anti–Gr-1 Ab may have also depleted a small proportion of Gr-1–positive monocytes in our model, due to the small decrease in the proportion of monocytes when examining the mononuclear cell populations (Table III). Previous studies have also reported a more proinflammatory and more severe phenotype following Gr-1 administration compared with Ly-6G administration, and it has been proposed that the nonneutrophil Gr-1–positive cells may have an important inhibitory role in regulating inflammatory signaling (27, 40). Future work identifying these nonneutrophil Gr-1–positive cell populations that may play an important role in limiting the inflammatory response will aid in the understanding of how the infection-induced inflammatory response of the uteroplacental tissues is regulated.

Although our data show that neutrophils appear not to be required for the onset of LPS-induced preterm labor in the mouse, the role of other immune cell populations known to influx in association with parturition remains unclear. Previous studies have reported that depletion of NK cells, invariant NKT cells, and macrophages attenuates LPS-induced preterm labor (20, 22, 47), whereas mice deficient in T and B cells are more susceptible to LPS-induced preterm labor (48). In our model, circulating lymphocytes and monocytes appear to have been largely unaffected by neutrophil depletion; therefore, these immune cell populations could be important in contributing to preterm labor in the neutrophil-depleted mice. Collectively, these studies highlight the complex interactions of different immune cells, and it is likely that different inflammatory stimuli will induce a different immune response, thus making depletion of a single immune cell population difficult to interpret in each case.

In summary, using a mouse model of LPS-induced PTL, we have demonstrated that the presence of an intrauterine infection induces a rapid neutrophil influx into the decidua. In contrast to our original hypothesis, the results of depletion experiments suggest that neutrophils are not required for the onset of LPS-induced preterm birth, but that they contribute to an LPS-induced inflammatory response of the uteroplacental tissues. Therefore, rather than being causal in the onset of PTL, it is likely that these infiltrating neutrophils are primarily involved in the postpartum repair and remodeling of the uterus and cervix. Further work is needed to confirm this hypothesis. Given that immune cell trafficking into the uteroplacental tissues has been identified as a key event in the onset of labor both at term and preterm, a clearer understanding of the role these immune cells play is critical to elucidate the underlying mechanisms involved in the initiation of parturition and to identify whether targeting specific immune cell populations may be an appropriate novel treatment approach for PTL.
